# Use of Virtual Reality for the Management of Anxiety and Pain in Dental Treatments: Systematic Review and Meta-Analysis

**DOI:** 10.3390/jcm9103086

**Published:** 2020-09-24

**Authors:** Nansi López-Valverde, Jorge Muriel-Fernández, Antonio López-Valverde, Luis Francisco Valero-Juan, Juan Manuel Ramírez, Javier Flores-Fraile, Julio Herrero-Payo, Leticia Alejandra Blanco-Antona, Bruno Macedo-de-Sousa, Manuel Bravo

**Affiliations:** 1 Department of Surgery, University of Salamanca, Instituto de Investigación Biomédica de Salamanca (IBSAL), 37007 Salamanca, Spain; nlovalher@usal.es (N.L.-V.); murimuriel@gmail.com (J.M.-F.); j.flores@usal.es (J.F.-F.); jhpayo@usal.es (J.H.-P.); lesablantona@gmail.com (L.A.B.-A.); 2 Department of Biomedical and Diagnostic Sciences, University of Salamanca, Avda. Alfonso X El Sabio S/N, 37007 Salamanca, Spain; luva@usal.es; 3 Department of Morphological Sciences, University of Cordoba. Avenida Menéndez Pidal S/N, 14071 Cordoba, Spain; jmramirez@uco.es; 4 Institute for Occlusion and Orofacial Pain Faculty of Medicine, University of Coimbra, Polo I-Edifício Central Rua Larga, 3004-504 Coimbra, Portugal; brunomsousa@usal.es; 5 Department of Preventive and Community Dentistry, Facultad de Odontología, Campus de Cartuja S/N, University of Granada, 18071 Granada, Spain; mbravo@ugr.es

**Keywords:** virtual reality, distraction systems, dental anxiety, pain

## Abstract

(1) Background: Dental treatments often cause pain and anxiety in patients. Virtual reality (VR) is a novel procedure that can provide distraction during dental procedures or prepare patients to receive such type of treatments. This meta-analysis is the first to gather evidence on the effectiveness of VR on the reduction of pain (P) and dental anxiety (DA) in patients undergoing dental treatment, regardless of age. (2) Methods: MEDLINE, CENTRAL, PubMed, EMBASE, Wiley Library and Web of Science were searched for scientific articles in November 2019. The keywords used were: “virtual reality”, “distraction systems”, “dental anxiety” and “pain”. Studies where VR was used for children and adults as a measure against anxiety and pain during dental treatments were included. VR was defined as a three-dimensional environment that provides patients with a sense of immersion, transporting them to appealing and interactive settings. Anxiety and pain results were assessed during dental treatments where VR was used and in standard care situations. (3) Results: 32 studies were identified, of which 8 met the inclusion criteria. The effect of VR in children was significant, both for anxiety (standardized mean difference (SMD) = −1.75) and pain (SMD = −1.46). (4) Conclusions: The findings of the meta-analysis show that VR is an effective distraction method to reduce pain and anxiety in patients undergoing a variety of dental treatments; however, further research on VR as a tool to prepare patients for dental treatment is required because of the scarcity of studies in this area.

## 1. Introduction

Pain suppression during dental interventions has been a major accomplishment for humankind. In 1842, William E. Clarke gave ether to a patient for the removal of a tooth; later, in 1844, a dentist named Horace Wells used nitrous oxide as an anesthetic for dental extractions; and in 1846, another dentist, William T. G. Morton, became a pioneer in the use of inhaled ether as an anesthetic at the Massachusetts General Hospital [1].

The anxiety associated with dental treatment is known as dental anxiety and certain authors have described it as the fifth most common cause of anxiety. It is determined by two circumstances: on the one hand, the prior act of anesthetizing, which in itself frequently causes a specific fear and, on the other hand, the subsequent dental treatment. Dental anxiety is a problem that dentists frequently face, being closely related to the painful stimulus and the increase in the threshold of pain perception, with the patients who suffer from it experiencing more pain, of longer duration, and with an exaggerated memory of its perception [2,3].

The number of studies on this pathology has exponentially increased over the last few years, growing from a very low number in the 1940s to the more than 6000 papers that are currently available, according to the U.S. National Library of Medicine [4].

Different therapies have been proposed for the prevention and treatment of pain (P) and dental anxiety (DA), among them virtual reality (VR) distraction techniques [5].

Although it is a concept that is difficult to define, VR is generally accepted as a three-dimensional environment generated by means of computer technology that creates a sense of immersion in the user, transporting the individual to appealing and interactive settings [6].

The benefits of using VR for the reduction of DA, P levels and dental phobia (a severe form of dental anxiety) during dental procedures has been extensively addressed in scientific literature [7,8,9,10,11], and its usefulness as a distraction tool is receiving increasing attention in medical contexts [11]. During aversive experiences, VR can improve pain management [12] and reduce the perceived duration of the procedure [13]. Moreover, a recent systematic review examined the effectiveness of virtual reality distraction in reducing pain [14]. This could be an advantage for many patients who reject DA control using anti-anxiety drugs because of their disadvantages or side effects, which can be, among others, impaired cognitive function and coordination, since they act as depressants on specific areas of the central nervous system [15,16,17].

The purpose of this study was to conduct a systematic review of literature comparing the effectiveness of the use of VR as a method for reducing anxiety and pain levels during dental treatment. This systematic review constitutes an essential tool to synthesize the scientific information available, increasing the validity of the conclusions and of individual studies, and identifying areas of uncertainty, where research is necessary. Meta-analysis (when possible) provides very useful information, to facilitate understanding of the effect of a treatment or intervention, both in general and in specific groups of patients. In addition, it allows us to increase the precision in the estimation of the effect, detecting effects of moderate magnitude, but of clinical importance, that could go unnoticed in primary studies.

## 2. Methods

The study selection process was carried out according to the Preferred Reporting Items for Systematic Review and Meta-Analyses (PRISMA) guidelines for systematic reviews and meta-analysis [18].

### 2.1. Protocol

The search strategy was conducted using the population, intervention, comparison and outcome (PICO) framework, based on the following question: 

“Are distraction techniques using VR effective against the anxiety and pain caused by dental procedures?”

To answer this question, a study population of patients who were undergoing dental treatment, with no age limit, was selected. The intervention consisted of using audio–visual or VR distraction methods. Controls were patients who were not subjected to audio-visual or VR distraction methods. The results revised in the literature were the DA or P values obtained using different validated scales:

1. For pain: Visual Analogic Scale (VAS), Wong–Baker Faces Scale (W–BFS) and Faces Pain Scale-Revised (FPS-R).
2. For anxiety: Consolability Scale (FLACC), Verbal Rating Scale (VRS), Modified Dental Anxiety Scale (MDAS), Corah’s Dental Anxiety Scale (CDAS) and Venham’s Clinical Anxiety Rating Scale (VCARS).

### 2.2. Search Method for the Identification of Studies

A search of the MEDLINE, CENTRAL, PubMed, EMBASE, Wiley Library and Web of Science electronic databases was conducted in November 2019 to identify relevant scientific articles. The search terms used were: “virtual reality”, “distraction systems”, “dental anxiety”, “pain”.

### 2.3. Inclusion and Exclusion Criteria

Inclusion criteria:

(a) Articles published in English.
(b) Parallel-arm randomized controlled clinical trials related to dental anxiety and pain associated with dental procedures in children and adults. For crossover clinical trials, only the first period or test-control comparison was considered.
(c) Standard care situations (dental anesthesia, dental extractions, dental fillings, mouth cleanings …)

Exclusion criteria:

(a) Non-randomized studies, non-controlled clinical trials or half-mouth design clinical trials.
(b) Comparative studies.
(c) Narrative reviews and systematic reviews.
(d) Case studies.
(e) Irrelevant and duplicate studies and those that did not meet the established inclusion criteria.

### 2.4. Data Extraction and Analysis

Studies that made no reference to the research question were removed, and the titles and abstracts of the articles selected were obtained and entered in an Excel spreadsheet. Two reviewers (N.L.-V. and J.M.F.) selected the titles and abstracts independently. Discrepancies in terms of study inclusion were discussed between the two aforementioned reviewers until consensus was reached. Subsequently, the full texts of the selected studies were obtained for their review and inclusion. For studies where more than one measure or scale for DA/P was used, we selected the more specific ones (for example, MDAS instead of pulse rate) with the most appropriate psychometric properties.

### 2.5. Risk of Bias (RoB) of Included Articles 

The Cochrane Collaboration, London, UK, tool was used to assess the methodology of the scientific evidence in all the selected studies [19].

### 2.6. Statistical Analysis 

Physiological data, such as pulse rate, degree of oxygen saturation, blood pressure and others were not included, incorporating only data related to pain and anxiety during dental treatment. Pain and anxiety were analyzed separately in children and adults. The mean scores and SDs (standard deviations) for pain and anxiety, during the procedure with VR and control, were extracted from the selected articles, using mean scores and interquartile ranges, or reported, directly, by the authors of the studies. Other information not related to VR was not taken into consideration in our meta-analysis. The different measurement scales and VR devices used were not considered. The meta-analysis was performed using Stata v.14.2 (StataCorp LP, College Station, TX, USA) and closely followed the methods proposed by the Cochrane Collaboration tool [19]. The methods can be observed in the different tables and figures. The standardized mean difference (SMD) was used as a measure of effect to account for different measurement scales both for anxiety and pain. Statistical heterogeneity among studies was assessed using the Q test according to Dersimonian and Laird and the I2 index (heterogeneity: I2 > 30% being moderate, >50% substantial and >75% considerable [19]). We decided to pool the study-specific estimates with the random effects model to protect our composite estimates (for anxiety and pain) from heterogeneity in the context of a relatively limited number of studies. We also decided a priori to present the results according to age group (children and adults) derived from the different clinical usefulness and interpretation. Finally, funnel graphs (not shown) and *p*-value calculation (Egger test) were used to assess publication bias.

## 3. Results

### 3.1. Characteristics of the Studies 

**Figure 1:**
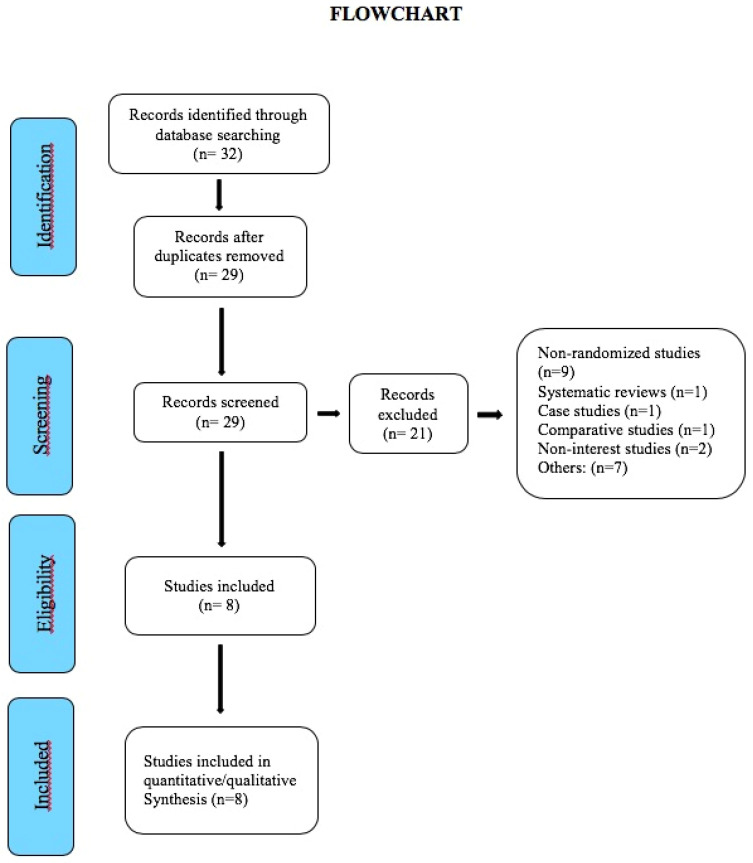
Flowchart of the study selection process. PRISMA (Preferred Reporting Items for Systematic Review and Meta-Analyses) [16].

Until November 2019, a total of 32 studies were gathered and subsequently assessed by the reviewers. Three duplicate studies were removed after an initial screening. A second screening, led to the removal of 14 studies, which left a total of 15 studies, and then we removed seven more: two for treating dental phobia through virtual reality exposure therapy (VRET) instead of distraction [8,9], one for being a protocol [20], one for not measuring DA/Pain as output, although DA is used as a predictor [11], two for being half-mouth [21,22], and one for lack of enough data for meta-analysis [23]. This yielded a final sample of eight studies for the analysis (Figure 1, Flowchart): seven in children [24,25,26,27,28,29,30] and one in adults [31]. It should be noted that six studies in children refer to both anxiety and pain [24,25,26,27,29,30], one in children only to anxiety [28], and the study in adults only to pain [31].

**Figure 2:**
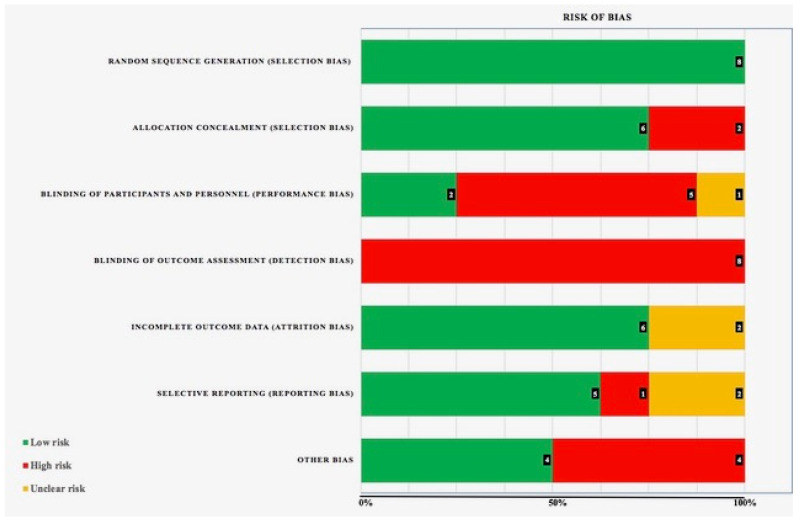
Risk of bias.

Table 1 provides a general description of the details of each study. The risk of bias (RoB Cochrane Collaboration Tool) in the studies considered is shown in Figure 2. All the studies complied with random sequence. It should be noted that none of the studies included complied with blinding of outcome assessment.

### 3.2. Virtual Reality (VR) and Anxiety Management 

**Figure 3:**
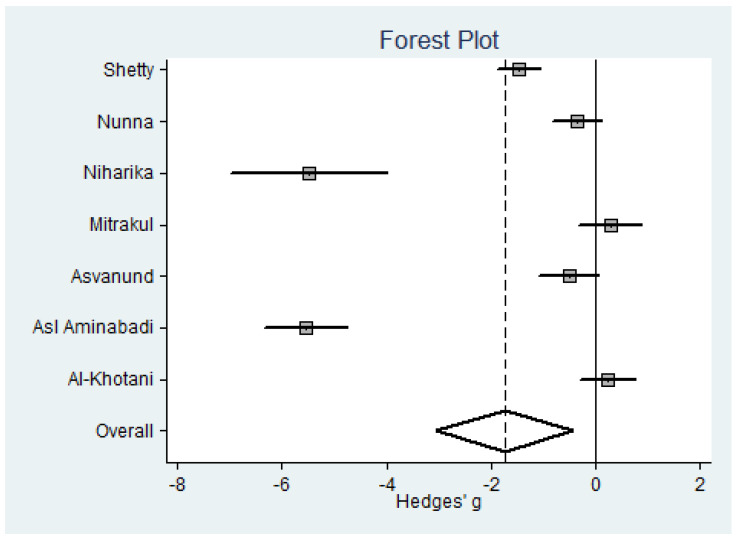
Forest plot for anxiety in children.

Regarding anxiety, the composite measure (data only for children) is significant, (SMD = −1.75, a substantial effect according to Cohen’s scale [32], *p* = 0.09) (Figure 3). Heterogeneity seems to be substantial (Table 2).

### 3.3. VR and Pain Management

**Figure 4:**
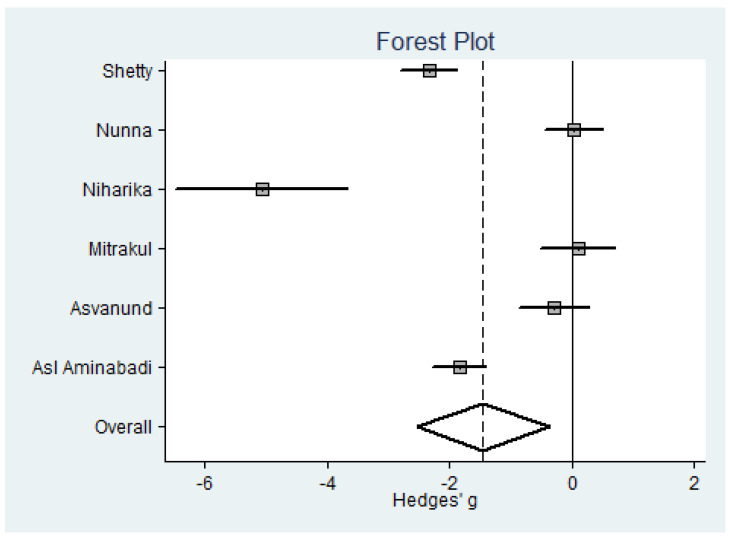
Forest plot for pain in children.

As regards pain, children are significantly protected (SMD = −1.46, i.e., a substantial effect according to Cohen’s scale [32] (Figure 4) (Table 2). In the case of adults, there is only one study [30] that reports a significant effect (SMD = 1.11, with 95% confidence interval (CI) = 1.71 to 0.51).

### 3.4. Publication Bias and Heterogeneity

None of the estimates seem to be affected by publication bias, according to the Egger test (Table 2).

## 4. Discussion

While anxiety and pain are usually associated with dental treatments, the number of studies addressing their management, especially during anesthetic block, which is one of the procedures that usually causes great anxiety among patients, is very limited [31,33]. 

In most studies are based on a pediatric population, fear of dental treatment affects 15–20% of the population, being recognized by the World Health Organization (WHO) as a real pathology [5,34,35] that leads those who are affected by it to reject even the most basic dental treatments, such as simple dental check-ups or cleaning [36]; thus, its management is essential to improve the patient’s quality of life [37]. 

Despite the existence of rigorous reviews of the literature [7], our systematic review and meta-analysis have focused on the efficacy of VR in pediatric patients (7 studies) and adults (1 study) who suffer anxiety triggered by dental treatments.

Our meta-analysis is based on 7 studies and has proved that VR is an effective tool for reducing dental anxiety (SMD = −1.75) and pain (SMD = −1.46) in children during a variety of dental procedures.

Some studies proved that, both for pain and anxiety, the use of VR was more effective in children than in adults. A possible reason for this could be that VR is especially appealing to children, since they become more engaged in whimsical thinking and are fascinated by imaginative play [38]. 

Nevertheless, regarding age, it should be noted that the differences in the efficacy of VR in each study could be due to the interpretive problems to which these analyses are susceptible; such phenomenon, known as ecological fallacies, could be associated either to the heterogeneity of the study’s characteristics (methodological diversity) or to the study population (clinical diversity) [39].

VR was found to be more effective for treating P and DA than conventional treatments; however, it is difficult to assess its efficacy as compared to other types of distraction. Klassen and colleagues [40] conducted a meta-analysis on distraction using music therapy as an alternative method to reduce anxiety and pain in different medical and dental procedures, finding a significant reduction with an effect size of 0.35. A Cochrane review of psychological interventions using different types of distraction to relieve pain in children and adolescents, published in 2006 and updated in 2013 and 2018 [41,42,43], reported different distraction techniques, such as musical therapy, reading, watching films, hypnosis, breathing techniques and combined cognitive-behavioral strategies, as effective tools to reduce pain and anxiety during needle procedures. However, the reviewers considered the level of certainty of the review to be low, since in most of these studies there was no blinding of participants and assessors. This is consistent with our meta-analysis, according to which only 35% of the studies included met this requirement.

The heterogeneity of VR software and hardware is also relevant to the immersive approach, which is influenced by the interaction with the virtual environment, either through translation or change of position, rotation or change of direction, viewpoint or perspective and visual field. This aspect is difficult to analyze when related to patients who are undergoing dental treatment (especially children), since adequate patient immersion is hindered by the fact that they are expected to remain with their heads as still as possible to facilitate the professional’s work [44]. 

Large devices (hardware), also hinder the dentist’s work, limiting vision of and access to the dental operation area [45].

Another interesting aspect that has not been given due regard by researchers is gender difference and its significance in terms of fear of dental treatment. Such differences have not been sufficiently addressed by researchers and clinicians and could contribute to greater effectiveness in the management of dental anxiety [46].

Likewise, patients’ different personality traits were not considered in the studies included either, whereas, moreover, none of the studies drew attention to factors that could moderate VR’s effectiveness, such as a subject’s sensitivity to anxiety or their temperament. Shy and emotional temperaments could be associated with dental anxiety [47,48,49,50,51].

Patients with dental fear and a high predisposition to anxiety magnify their pain expectations when they are exposed to critical situations. When patients with dental anxiety undergo dental treatment, their beliefs about the negative consequences of bodily excitement can negatively influence their assessment of pain linked to such treatment [52]. DA as a predisposing factor is associated with a state of anxiety, which has a constant impact on pain during the patient’s entire dental treatment; hence, anxiety should be assessed as a critical step not only towards anxiety management in patients with high DA but also towards P management in all dental patients [53].

On the other hand, the studies included used different pain and DA assessment scales: VAS, W-BFS, FPS-R FLACC, VRS, MDAS, CDAS and VCARS. 

Likewise, none of the studies included single scales for joint assessment by dentist and patient. Vital signs were also assessed in some of the studies as emotional state indicators. Accordingly, it would be convenient and appropriate to find validated scales that might be used to jointly assess all of these aspects. In a systematic review of 32 studies, Astramskaitė and Juodžbalys did not find an adequate scale to assess the patient’s stress, pain and fear at the same time, and propose a questionnaire that is suitable only for dental extractions [54].

Eijlers and colleagues [7,55], presented two preparatory studies for pediatric surgery based on VR training programs; however, the use of this type of preparatory program, before certain dental procedures, is yet to be explored by researchers and, therefore, it is currently not possible to compare effects with and without preparation. 

For all these reasons, we believe that this systematic review has certain limitations in terms of number, quality and methodology of the studies included.

Hence, to determine the effects of VR distraction on anxiety and pain in dental treatments, it would be necessary to reduce the risk of bias, to remove confusion factors and to establish a clear definition of the adequate parameters, all with the purpose of obtaining results that can be translated into broad clinical applications, so that evidence can effectively support the practice of clinical dentistry.

## 5. Conclusions

This systematic review and meta-analysis lead to the conclusion that VR is a useful tool to reduce DA and P in children undergoing dental treatment.

No significant effect was found for DA. Studies in adults are scarce. On the other hand, most of the studies chose to focus on immersion in the pediatric population, neglecting a series of aspects that should be considered, such as training programs, the different types of software and hardware of virtual reality devices, patient temperament and personality, type of treatment, gender difference and more. Due to all this, the role of virtual reality in the control of dental anxiety and pain in children and adults should be considered as a topic for future research.

## Figures and Tables

**Table 1:** Details of each study.

Study (Year)	Journal	Children Values (Ma, *n*, Ar)	Adult Values (Ma, *n*, Ar)	Dental Procedure	Virtual Reality (VR) Device Equipment	Measuring Scales		Outcomes
						DA	*p*	
Niharika et al. 2018 [24]	J Indian Soc Pedod Prev Dent	Ma = Group A (7.17 ± 0.316) Group B (7.28 ± 0.300) *n* = 40 Ar = 4–8		Routine dental care (pulp therapy in mandibular primary molars). Local anesthetic.	Google VR Box and Anti-Tank Virtual Reality 3D Glasses	MDAS	W-BFS	Two groups. Childhood Anxiety-Related Disorders scores did not differ significantly between the two groups. In both groups, a statistically significant difference was detected between the two treatment sessions (with and without VR).
Asl Aminabadi et al. 2012 [25]	J Dent Res Dent Clin Dent Prospect	Ma = 5.4 *n* = 120 Ar = 4–6		Restorative treatment in primary molars.	I-glasses 920HR Ilixco, Inc. Menlo Park, CA, USA.	MDAS	W-BFS	There was a significant decrease in pain perception and anxiety scores with the use of VR eyeglasses during dental treatment.
Nunna et al. 2019 [26]	J Dent Anesth Pain Med	Ma = Nr *n* = 70 Ar = 7–11		- Counter-stimulation. - Local anesthesia administration with virtual reality distraction.	Lenovo smartphone, Sennheiser earphones, and ANTVR glasses.	VCARS	W-BFS	Assessment of mean anxiety scores showed a significant difference in girls belonging to the VR group.
Shetty et al. 2019 [27]	The Journal of Clinical Paediatric Dentistry	Ma = Nr *n* = 120 Ar = 5–8		Dental treatment (vital pulp therapy)	Eyeglasses. VR device (i-glasses 920HR, Ilixco Inc., Menlo Park, CA, USA)	MDAS	W-BFS	Two groups. The group where VR distraction was used reported a decrease in the severity of anxiety. Lower pain scores were observed in the VR group.
Al-Khotani et al. 2016 [28]	Acta Odontologica Scandinavica	Ma = 8.2 *n* = 56 Ar = 7–9		Dental examination, oral hygiene information, prophylaxis, restorative treatment.	Eyeglasses. DVD Players, gaming systems like Sony Play Station Pro, Microsoft X-BOX, Nintendo WII	MDAS		Two groups. VR and control group. Significant reduction in anxiety throughout the restorative procedure (including injection with local anesthesia) in VR group.
Mitrakul et al. 2015 [29]	European Journal of Paediatric Dentistry	Ma = 6.9 ± 0.9 *n* = 42 Ar = 5–8		Restorative dental treatment in maxilla or mandible under local anesthetic injection.	Eyeglasses. (Shenzhen Longway Vision Technology Co. Ltd., Shenzhen, China).	FLACC	FPS-R	Two groups. Group 1 received treatment without wearing VR during the first visit and wearing VR during a second visit. In Group 2, VR was used viceversa.
Asvanund et al. 2015 [30]	Quintessence International	Ma = 7 ± 0.8 *n* = 49 Ar = 5–8		Restorative dental treatment (local anesthetic injection in the maxillary arch or mandibular block).	Eyeglasses (Shenzhen Longway Vision Technology Co. Ltd., Shenzhen, China).	FLACC	FPS-R	Two groups. The study assesses pain and anxiety without distinction. The limitation of this study is that the FLACC score was assessed by playing back the video recording of each visit, which was done by two pediatric dentists who could not be blinded to the child’s use of VR.
Sweta et al. 2019 [31]	Ann Maxillofac Surgery		Ma = 39.72 ± 15.93. *n* = 50 Ar = Nr	Local anesthesia in patients undergoing a dental procedure.	Nr	CDAS		Local anesthesia and extractions reported the highest anxiety levels among the patients. Limitations of this study: - Small sample size. - Patients were not in control of their VR environment.

*n,* Participant number; Ma, Mean age years; Ar, Age range years; Nr, Not reported; FLACC, Consolability Scale; MDAS, Modified Dental Anxiety Scale; VCARS, Venham’s Clinical Anxiety Rating Scale; CDAS, Corah’s Dental Anxiety Scale; W–BFS, Wong–Baker Faces Scale. DA, Dental anxiety; P, Pain.

**Table 2:** Meta-analysis of anxiety and pain in children. Characteristics of individual studies and meta-analysis.

			Test		Control			SMD ^a^			Heterogeneity I^2^ (*p*-Value)	Public. Bias *p*-Value (Egger Test)
Age group/Study	Year	Scale	*n*	mean ± sd	*n*	mean ± sd	Weight	Mean	95% CI	*p*-value		
Anxiety (*n* = 7) (see Figure 3)												
Shetty [27]	2019	MDAS	60	11.3 ± 3.5	60	16.5 ± 3.5	14.8%	−1.48	−1.88 to −1.07			
Nunna [26]	2019	VCARS	35	0.57 ± 0.61	35	0.8 ± 0.7	14.7%	−0.35	−0.83 to 0.12			
Niharika [24] ^b^	2018	MDAS	18	14.7 ± 0.8	18	19.6 ± 0.9	12.6%	−5.48	−6.97 to −3.99			
Mitrakul [29] ^b^	2015	FLACC	21	0.95 ± 1.63	21	0.57 ± 0.98	14.5%	0.28	−0.33 to 0.89			
Asvanund [30] ^b^	2015	FLACC	26	1.65 ± 2.04	23	2.7 ± 2.0	14.6%	−0.51	−1.08 to 0.06			
Asl Aminabadi [25] ^b^	2012	MDAS	60	12.6 ± 1.0	60	18.2 ± 1.0	14.2%	−5.55	−6.35 to −4.75			
Al-Khotani [28]	2016	MDAS	28	1.93 ± 1.15	28	1.68 ± 0.86	14.6%	0.24	−0.28 to 0.77			
Total			248		245		100%	−1.75	−3.06 to −0.43	0.009	51% (*p* = 0.058)	*p* = 0.197
Pain (*n* = 6) (see Figure 4)												
Shetty [27]	2019	W-BFS	60	2.42 ± 1.47	60	5.60 ± 1.22	17.4%	−2.34	−2.81 to −1.87			
Nunna [26]	2019	W-BFS	35	3.03 ± 2.02	35	2.97 ± 2.49	17.3%	0.03	−0.44 to 0.49			
Niharika [24] ^b^	2018	W-BFS	18	2.56 ± 0.39	18	5.44 ± 0.68	13.8%	−5.07	−6.47 to −3.67			
Mitrakul [29] ^b^	2015	FPS-R	21	1.90 ± 2.93	21	1.62 ± 2.94	17.0%	0.09	−0.51 to 0.7			
Asvanund [30] ^b^	2015	FPS-R	26	2.23 ± 2.29	23	3.04 ± 3.08	17.1%	−0.30	−0.86 to 0.27			
Asl Aminabadi [25] ^b^	2012	W-BFS	60	1.89 ± 0.65	60	3.05 ± 0.60	17.4%	−1.84	−2.27 to −1.41			
Total			220		217		100%	−1.46	−2.54 to −0.37	0.008	49% (*p* = 0.082)	*p* = 0.617

^a^: Standardized difference of means. ^b^: This is a cross-over clinical trial. We have considered only the comparison test-control in the first period. MDAS, Modified Dental Anxiety Scale; VCARS, Venham’s Clinical Anxiety Rating Scale; FLACC, Consolability Scale; W-BFS, Wong–Baker Faces Scale; FPS-R, Faces Pain Scale-Revise; CI, Confidence Interval.

## Abbreviations

VR	Virtual Reality
DA	Dental Anxiety
VAS	Visual Analogic Scale
W–BFS	Wong–Baker Faces Scale
FPS-R	Faces Pain Scale-Revise
FLACC	Consolability Scale
VRS	Verbal Rating Scale
MDAS	Modified Dental Anxiety Scale
CDAS	Corah’s Dental Anxiety Scale
VCARS	Venham’s Clinical Anxiety Rating Scale
RoB	Risk of Bias
SDs	Standard Deviation
SMD	Standard Mean Deviation
CI	Confidence Interval
